# Perspectives of parents of working adolescents in Ontario, Canada

**DOI:** 10.1186/s12889-021-10377-9

**Published:** 2021-02-09

**Authors:** Viswanathan Shankar, Carol W. Runyan, Scott B. Harpin, John Lewko

**Affiliations:** 1 Department of Epidemiology and Population Health, Albert Einstein College of Medicine, 1300 Morris Park Ave, Block Building Rm 315, Bronx, NY 10461 USA; 2 Department of Epidemiology and Program for Injury Prevention, Education and Research, Colorado School of Public Health, Aurora, CO USA; 3 University of Colorado College of Nursing, Aurora, CO USA; 4 School of Rural and Northern Health, Laurentian University, Sudbury, Canada

**Keywords:** Adolescents, Occupational injury, Parent, Workplace, Safety

## Abstract

**Background:**

More than half of adolescents have jobs in summer or sometime during the year. While employers are ultimately responsible for their safety, parents are often important in helping their children navigate the work environment. Our study examines the attitudes, beliefs and types of involvement parents have in their children’s work.

**Methods:**

We modeled a telephone survey of 507 English-speaking parents of working adolescents in Ontario, Canada on a US study and examined their perspectives, comparing to earlier findings from the U.S. parents.

**Results:**

Most Ontario parents helped their teens consider questions to ask about work, for example, work hours (90.7%) and job tasks (78.2%) and fewer about workplace safety (57.9%). Parents overall were concerned about their teens, especially younger teens, getting behind on schoolwork (69.3%), being rushed on the job (60.1%) and doing hazardous tasks (58.3%) or working alone (51.9%), or being at work during a robbery (74.5%). Parents of 14–17-year-old daughters were more concerned about their child being assaulted than were parents of sons (62.4% vs. 51.4%), particularly if the teen was in the 18–19 age group (74.3% vs. 52.5%). Half the parents indicated 10–19 h per week was the right amount of work time for their teen, and most agreed that laws should limit the number of hours of youth work.

**Conclusions:**

Overall, Ontario parents appear to be more concerned about the safety and also more involved in the work of their adolescent children than U.S. parents previously surveyed. Parents are engaged with their children about their work and may serve as valuable assets to helping to advocate for safe work policies and environments.

**Supplementary Information:**

The online version contains supplementary material available at 10.1186/s12889-021-10377-9.

## Background

According to the Statistics Canada Labor Force Survey Estimates [1], approximately 983,000 young people aged 15–24 years old living in the province of Ontario were employed between June and October 2019. This compares to 972,000 Ontario youth (ages 15–24) working in 2008 between June and October 2008 [1] when we collected the data reported here, or 56% of youth in that age range [2], slightly less than the proportions working in the US (62%) at the time of our US analysis [3] While there currently are proportionally more full-time workers and higher rates of employment during the summer months, that number decreased during the autumn months to about a 49:51 full-time to the part-time ratio in the month of October. In a trend analysis of all Canadian youth between 2008 through 2014, Bernard found 49.2% of Ontario youth ages 15–19, and 73.4% of those in the 20–24 age group were working in 2014 [3]. The great majority of those jobs are in sales and service occupations—as in the United States [4].

The Ontario Ministry of Labour reports that 6000 young workers were injured seriously enough each year to miss work, and 17 young workers between ages 15 and 24 experienced fatalities between 2010 and 2015 [5]. Canadian hospital data examined by Pratt et al., revealed higher incidences of injuries in the retail and service sectors, that males were more frequently injured than females, and that young workers experienced higher rates than their adult counterparts [6].

Though legally the responsibility for a safe workplace falls to employers, parents are a potential source of help to young workers navigating the work environment. A national survey of parents of U.S. working teens, [7] observed that American parents, although generally aware of the presence of child labor laws, were ill-informed about specific labor and safety restrictions, with fewer than 15% correctly answering questions about hour restrictions, fewer than a quarter being aware of restrictions on motor vehicle use by teens under age 18, and only about half knowing about restrictions on the use of forklifts or power slicers. The majority of parents in the same survey reported that they helped their teenagers find job opportunities and complete applications or prepare for interviews, met the youth’s supervisor, or had visited the worksite. However, few reported helping youth learn about their rights as a worker, about work restrictions, or how to get the proper job training [8]. Parents in the US expressed concern about a number of the job duties their children were performing, yet overall were favorable to their adolescents working [9].

Despite large numbers of teens and young adults working in Canada, less attention has been paid to the role of Canadian parents concerning the work of their teenage children [10]. In 2014, Usher and colleagues conducted focus groups with 34 parents of 12–14-year old urban Ontario workers to understand their perceptions of work hazards [11]. Parents of these young workers described many benefits of such paid work and suggested the benefits outweigh the risks. Parents reported more vigilance when their child was working in a self-employed situation (e.g., shoveling snow, babysitting) than in a more traditional workplace. They did express particular concerns about assault and kidnapping.

Our population-based study was designed to examine the knowledge, attitudes, and behaviors of Ontario parents regarding working adolescents (age 14–19), an age demographic that performs a wide range of occupational tasks with potential hazards. We also compare parent responses with those from earlier work in the U.S., where there are specific child labor laws [7–9].

## Methods

During 2008, we contracted the Waterloo Survey Research Centre to conduct computer-assisted telephone interviews with parents or guardians (referred to hereon as “parents”) of working adolescents, ages 14–19, using a 125 item instrument adapted from a US study [9]. The survey instrument was modified from the US version to reflect Canadian labor laws. The parent questionnaire is provided as supplemental material. The sample came from the ICOM Target Source Canada survey database, which included people who had previously participated in a national household survey and agreed to be re-contacted. The original sample had been selected to be representative of Ontario demographics [4]. Making up to 12 call attempts per household, interviewers offered gift cards to those who completed the survey. Details of our survey procedures appear in an earlier paper by Lewko et al. [4].

Respondents had to be English-speaking parents of an adolescent (age 14–19), who worked in the service sector for at least 2 months in the prior year. Parents consented verbally, as approved by the Research Ethics Board (REB) at Laurentian University and by the Office of Research Ethics (ORE) at the University of Waterloo.

Sampling weights for parents were estimated by a two-step process by first developing (base) design weights of each participating parent based on the inverse probability of selection of the household within the frame. In the second step, we made a post-stratification adjustment to the sample weights, based on the region (CMA Toronto versus rest of Ontario), household income, and gender of teens between ages 13 and 17 using the 2006 Census for calibration. We computed descriptive statistics for parents’ attitudes and beliefs regarding their children’s work, adjusting for their sampling weights. The weighted proportions and corresponding logit transformation based 95% confidence intervals are presented. We further examined the distribution of parental involvement and concerns in teens’ work by teen’s gender, performing an unconditional subclass analysis (domain analysis) by the teen worker’s age (14–17 and 18–19 years) and parents’ gender.

For selected variables, we present comparison estimates from U.S. parentally-reported data obtained in 2003 about adolescents working in the retail and service sector [7–9]. All analyses were done using Stata software version 14.0.

## Results

### Sample

The survey reached 534 Ontario households, of which 507 (94.9%) parents participated. Most were mother (78.3%) or father (19.1%) figures of the young workers, with the remainder being a mix of other relatives (2.7%). Fifty-eight percent were Caucasian, and of those who responded, the median annual household income was between $80,000 and $89,000 (Canadian).

### Parental involvement in teen work

As with the U.S. sample,^7^ most Ontario parents reported being engaged with their teens in various aspects of the employment process, including helping them find job opportunities (89.1%) and preparing for interviews (79.7%) to considering questions to ask about job tasks (78.2%) (Table 1). Most parents helped their teens consider questions about work hours (90.7%), but fewer addressed workplace safety (57.9%). More than two-thirds of Ontario parents had met the direct supervisor, and more than half had visited the worksite. In both the US and Ontario samples, higher proportions of parents of female than male workers were engaged in activities like visiting the workplace, asking about working conditions, and meeting with the supervisor. Regardless of the gender of the teen worker, higher proportions of Ontario parents, compared to US parents, were engaged in asking about workplace safety issues and learning about workers’ rights. We found differences in parental involvement in tasks such as filling out job applications based on whether the child was male vs. female (86.7% vs. 77.8%) and helping the teens consider questions to ask the employer about workplace safety (52.7% vs. 62.7%). Comparing by parental gender (Supplemental Table 1), we discovered that fathers and mothers reported equal engagement in helping both sons and daughters fill out applications. Fathers were more involved in helping their sons vs. daughters handle difficult issues other than safety (70.3% vs. 48.2%) while more mothers addressed these issues with their daughters vs. sons (72.5% vs. 63.3%). Fathers were more likely to encourage sons to quit a job because of concerns the teen would get injured on the job (19.5%) compared to doing so with only 2.7% of their daughters. However, mothers reported counseling about the same portion of sons vs. daughters about quitting a job because of safety concerns (15.8% vs. 14.9%).

**Table 1:** Parental involvement in teen work by gender of worker, Ontario (2008) and United States (2003)

	Gender of teen worker - Ontario			Gender of teen worker – United States^a^	
	Male (***n*** = 244) % (95% CI)	Female (***n*** = 263) % (95% CI)	Total (507) % (95% CI)	Male (***n*** = 480) % (95% CI)	Female (***n*** = 442) % (95% CI)
**Consider questions to ask about work hours?**	89.9 (84.3, 93.6)	91.5(87.3,94.4)	90.7 (89.4, 93.2)	87.2 (80.8–93.5)	90.0 (85.9–94.2)
**Identify job opportunities**	88.9 (83.2, 92.8)	89.2 (82.3, 93.6)	89.1 (84.8, 92.2)	87.5 (81.5–93.5)	91.8 (88.3–95.3)
**Fill out job applications**	86.7 (80.4, 91.1)	77.8 (69.4, 84.4)	82.0 (76.9, 86.2)	79.8 (73.7–86.0)	84.6 (80.3–89.0)
**Prepare for a job interview**	83.8 (77.0, 88.8)	76.0 (67.3, 83.0)	79.7 (74.2, 84.3)	75.1 (68.4–82.1)	80.4 (75.4–85.5)
**Consider questions to ask about job tasks**	79.5 (73.0, 84.8)	76.9 (68.5, 83.6)	78.2 (72.9, 82.6)	75.3 (68.4–82.2)	82.6 (77.6–87.5)
**Met direct supervisor**	68.8 (60.7, 75.9)	70.9 (63.2, 77.5)	69.9 (64.3, 74.9)	79.7 (73.8–85.7)	83.5 (787–88.4)
**Handle difficult issues other than about safety**	64.6 (56.9, 71.6)	67.6 (59.0, 75.1)	66.2 (60.4, 71.4)	70.1 (63.2–77.0)	69.8 (62.8–76.8)
**Helped teen get more training to do a job**	63.9 (56.5, 70.7)	63.1 (55.7, 70.0)	63.5 (58.4, 68.3)	37.8 (30.5–45.1)	39.3 (31.3–47.3)
**Visited workplace to monitor conditions**	58.7 (50.8, 66.1)	67.5 (58.9, 75.0)	63.3 (58.4, 68.3)	58.4 (50.7–66.1)	67.6 (60.6–74.5)
**Helped teen learn about worker’s rights**	58.0 (50.4, 65.3)	61.1 (52.8, 68.7)	59.6 (54.0, 64.9)	45.4 (37.7–53.0)	51.8 (43.9–59.7)
**Consider questions to ask about workplace safety**	52.7 (45.0,60.3)	62.7 (54.7, 70.1)	57.9 (52.3, 63.3)	44.6 (37.1–52.2)	47.9 (39.9–56.0)
**Encouraged teen to report a violation about teen’s work to a government agency?**	17.5 (12.2, 24.5)	19.4 (13.1, 27.6)	18.5 (14.2, 23.7)	n/a	n/a
**Encouraged teen to quit a job because you were concerned about teen getting injured on the job?**	16.5 (11.2,23.5)	12.4 (7.4, 20.1)	14.4 (10.6, 19.2)	n/a	n/a

^a^ = U.S. results previously reported in: [8].

*n/a* not asked in the US survey

### Ontario parental concerns about work safety

Though not shown in the tables, most (85.8, 95% CI: 80.7, 89.8) of the Ontario parents reported being somewhat or very familiar with problems their teens faced at work, and 86.8% (95% CI: 82.5, 90.1) reported being at least somewhat involved in giving their teen advice about work. When asked whether they considered any aspects of their teen’s job to be hazardous, 69.4% (95% CI: 64, 74.3) said “no”.

As shown in Table 2, parents overall were concerned about their teens, especially younger teens, getting behind on schoolwork (69.3%; 95% CI: 64.3, 73.9), being rushed on the job (60.1%; 95% CI: 54.5, 65.5) and doing hazardous tasks (58.3%; 95% CI: 52.7, 63.7) or working alone (51.9%; 95% CI: 46.1, 57.6) or being at work during a robbery (74.5%; 95% CI: 69.4, 79.0).

**Table 2:** Ontario parents’ (*n* = 507) compared to US parents’ (*n* = 1053) concerns about adolescent work by age and sex of working adolescent

	U.S. Parents		Ontario Parents				
Parents indicating “very concerned” or “somewhat concerned”	Age of worker 14–17 years^a^		Age of worker 14–17 years (*n* = 408)		Age of worker 18–19 years (*n* = 99)		All ages
	Male (***n*** = 544) % (95% CI)	Female (***n*** = 509) % (95% CI)	Male (***n*** = 199) % (95% CI)	Female (***n*** = 209) % (95% CI)	Male (***n*** = 45) % (95% CI)	Female (***n*** = 54) % (95% CI)	Total (***n*** = 507) %(95% CI)
Not using protective equipment or clothing	41.4 (33.7, 49.1)	31.4 (23.7, 39.1)	60.6 (52.1, 68.5)	36.9 (28.4, 46.2)	63.6 (46.1,78.0)	53.2 (35.2, 70.3)	50.0 (44.3, 55.7)
Working late at night	34.5 (27.4, 41.5)	38.5 (31.2, 45.8)	55.6 (47.0, 63.8)	55.0 (45.8, 63.9)	62.5 (45.1, 77.1)	66.2 (48.7, 80.1)	56.9 (51.3, 62.4)
Not having safety training to do and complete job tasks safely	51.3 (43.6, 59.0)	44.7 (36.8, 52.7)	63.9 (55.6, 71.4)	47.3 (38.0, 56.7)	72.8 (55.4, 85.1)	62.2 (44.8, 77.0)	57.4 (51.7, 62.9)
Not having safety training to identify risky & hazardous conditions on the job	n/a	n/a	60.8 (52.2, 68.8)	50.5 (41.2, 59.7)	70.9 (52.9, 84.1)	73.2 (57.4, 84.7)	58.5 (52.8, 64.0)
Not having received adequate training.	n/a	n/a	53.4 (44.8, 61.7)	47.9 (38.7, 57.2)	55.0 (37.2, 71.7)	62.9 (45.8, 77.4)	52.1 (46.3, 57.7)
Not getting enough sleep because of his/her job	38.1 (30.6, 45.6)	40.8 (33.2, 48.4)	58.0 (49.4, 66.0)	54.8 (45.4, 63.9)	56.0 (38.0, 72.5)	69.2 (53.0, 81.7)	57.5 (51.7, 63.0)
Getting behind in school work because of his/her job	42.6 (35.3, 48.9)	47.6 (39.8, 55.3)	73.6 (65.4, 80.4)	68.6 (60.8, 75.4)	55.2 (37.3, 71.9)	68.7 (51.7, 81.9)	69.3 (64.3, 73.9)
Being rushed on the job.	30.9 (23.7, 38.0)	38.0 (30.2, 45.8)	63.1 (54.6, 70.8)	54.9 (45.5, 63.9)	72.1 (55.2, 84.4)	60.2 (42.6, 75.5)	60.1 (54.5, 65.5)
Handling hazardous equipment, chemicals or toxic substances.	34.3 (26.4, 42.2)	24.3 (17.3, 31.4)	55.2 (46.6, 63.4)	38.0 (29.2, 47.7)	53.0 (35.1, 70.2)	54.1 (36.2, 70.9)	47.5 (41.8, 53.3)
Doing hazardous tasks.	35.0 (27.3, 42.8)	29.7 (22.0, 37.4)	65.5 (56.9, 73.1)	47.1 (38.1, 56.3)	67.5 (49.2, 81.6)	71.5 (56.3, 83.0)	58.3 (52.7, 63.7)
Working alone.	38.5 (30.6, 46.4)	42.1 (34.4, 49.8)	53.8 (45.3, 62.0)	47.9 (36.3, 70.9)	54.1 (36.3, 70.9)	60.0 (42.5, 75.4)	51.9 (46.1, 57.6)
Getting physically or sexually assaulted.	26.0 (19.8, 32.2)	46.8 (39.0, 54.5)	51.4 (42.8, 59.8)	62.4 (53.2, 70.8)	51.5 (33.8, 68.9)	74.3 (59.0, 85.3)	58.2 (52.7, 63.6)
Being at work during a robbery.	46.3 (38.7, 54.0)	54.5 (46.8, 62.1)	67.2 (58.6, 74.8)	78.4 (70.2, 84.9)	76.2 (60.9, 86.8)	85.2 (72.5, 92.7)	74.5 (69.4, 79.0)

^a^ = U.S. results previously reported in: [9].

*n/a* = not asked in the US survey

Fewer than a quarter (23.7, 95% CI: 19.2, 28.9), however, indicated that it was likely that their child would be injured seriously enough to “need medical attention or … miss one or more days of school or work during the next 12 months”, with parents of males vs. female working teens more likely to expect an injury (28.1% vs. 19.7%) respectively.

Approximately 3 in 10 (27.6, 95% CI: 23.0, 32.8) of Ontario parents indicated that any of their child’s tasks or working conditions were hazardous and identified a wide variety of specific concerns, including working around hot surfaces (23.9, 95% CI: 16.8, 32.9), exposure to hot liquids or grease (22.8, 95% CI: 15.5, 32.3), using sharp cutting instruments such as case cutters or knives (19.8, 95% CI: 13.4, 28.4), moving heavy objects (15.1, 95% CI: 9.7, 22.8) or encountering slippery floors (11.8, 95% CI: 6.7, 19.8). Fewer than 6% expressed concern about any of the following potential hazards: powered machinery, falling objects, cluttered and crowded workplaces, working where heavy equipment is operating, or exposure to needles, blood products, or medical waste. This is in the context of 47.8% (95% CI: 42.3, 53.4) having indicated their child had used sharp instruments, 33.8% (95% CI:28.5, 39.5) saying their adolescents’ had done heavy lifting at work, and 12.2% (95% CI:9.1, 16.2) reporting their child had used a power slicing device or grinder.

At least 35% of Ontario parents expressed concern about each of the 13 issues we examined related to their adolescents’ work (Table 2). Though varying somewhat by the age and gender of the youth, a large percentage expressed concern about their child being at work during a robbery, and more than 64% of the parents of daughters reported concerns about physical or sexual assault. We observed differences among parental concerns for their working sons vs. daughters in the age 14–17 group; for example, not using protective equipment or clothing (60.6% vs. 36.9%), not having safety training (63.9% vs. 47.3%), handling hazardous equipment, chemical or toxic substances (55.2% vs. 38.0%), and being at work during a robbery (67.2% vs. 78.4%).

The majority of parents of younger male workers and both male and female workers in the 18–19 age range were concerned about their child doing hazardous tasks and being exposed to hazardous equipment, chemicals or other toxic substances. Parents also worried about the effects of work on their child’s schoolwork. The majority of parents of working males, regardless of age, and of females in the 18–19 age range, were concerned about safety training for the identification of risky and hazardous conditions and for completing job tasks safely.

As shown in supplemental Table 2, when assessed by the parental gender, overall, both mothers and fathers expressed more concern about their sons vs. daughters not using protective equipment, not having safety training (to do or complete tasks, and doing hazardous tasks). Parents overall expressed more concern about their female children working during a robbery or getting physically or sexually assaulted than their male children.

### Ontario parents’ views on work hours

When asked about the maximum number of hours a worker under age 18 and still in school should be allowed to work during a week when school is in session, about 52.9% indicated they thought 10–19 h, with 16.9% indicating teens should work fewer than 10 h and 30.3% stating the maximum should be 20 or more hours.

As shown in Table 3, more than 39% of Ontario parents expressed a belief that adolescents under age 16 should not work past 8 PM on a night when there is school the next day, and another 43% suggested 9 PM as the latest stopping time on school nights. For older youth, parents were more agreeable to later hours. Nearly 98% believed that children 16–17 years should complete work at the latest by 11 PM. There was no difference observed when compared by gender of the teen or the parent (supplemental Table 3). This is similar to the percentages of US parents (not shown) who indicated that work hours should not exceed 20 per week during the school year; however, more than a third of US parents were receptive to allowing their teens to work 20 h or more per week during the school year [9].

**Table 3:** Beliefs of Ontario parents about teen work hours when the working teens are attending school, stratified by gender of teen, 2008 (*n* = 507)

Teen Gender	Male (***n*** = 244), % (95% CIs)	Female (***n*** = 263), % (95% CIs)	Total (***n*** = 507) % (95% CIs)
**In your opinion, what is the latest hour that a teen worker UNDER 16 should be allowed to work when there is school the next day?**			
Earlier than 8 PM	14.4 (9.7, 20.8)	18.5 (12.5, 26.7)	16.5 (12.5, 21.6)
8 PM	25.5 (18.7, 33.8)	19.2 (13.5, 26.5)	22.2 (17.6, 27.6)
9 PM	41.0 (33.9, 48.6)	43.7 (35.7, 52.0)	42.4 (36.9, 48.1)
10 PM	13.9 (9.82, 19.2)	14.8 (9.91, 21.6)	14.3 (11.0, 18.6)
11 PM	2.4 (1.1 5.1)	1.6 (0.6, 3.2)	1.95 (1.07, 3.51)
12 AM or later	0.6 (0.1, 2.5)	0.4 (0.05, 2.5)	0.50 (0.15, 1.51)
Refused or Don’t Know	2.3 (0.8, 5.9)	1.9 (0.6, 5.2)	2.05 (0.99, 4.18)
**In your opinion, what is the latest hour that a 16–17 year old should be allowed to work when there is school the next day?**			
Earlier than 8 PM	3.0 (1.4, 6.0)	7.5 (3.2, 16.8)	5.3 (2.7, 10.1)
8 PM	6.1 (3.3, 10.9)	8.1 (3.9, 115.4)	7.1 (4.4, 11.3)
9 PM	40.2 (32.7, 48.3)	32.3 (25.4, 39.9)	36.1 (30.8, 41.7)
10 PM	35.3 (28.5, 42.9)	38.3 (30.5, 46.8)	36.9 (31.6, 42.6)
11 PM	12.4 (8.5, 17.9)	10.7 (7.4, 15.2)	11.5 (8.8, 14.9)
12 AM or later	2.1 (0.8, 5.2)	2.1 (0.9, 4.6)	2.1 (1.2, 3.8)
Refused or Don’t Know	0.7 (0.1, 5.3)	1.1 (0.3, 3.0)	0.9 (0.3, 2.5)

### Parental attitudes and beliefs about controlling work for teens

Although not shown in the table, parental views on governmental restrictions of late-night hours are more varied. While 14.2% of US parents endorsed the statement “laws that keep teenagers from working late at night on school nights are a bad idea,” 22.7% of Ontario parents agreed with the statement. In Ontario, 77.5% of parents agreed that “laws should limit the number of daily and weekly hours that teenagers can work,” compared to 85% of US parents [8].

In terms of other specific controls on teen work, similar to US parents, virtually all Ontario parents (> 97%) supported the importance of safety training, the use of safety clothing, and of safety equipment as well as having qualified supervisors. To the same degree, they uniformly supported the statements that parents “should look out for safety issues” as well as the statement that “employers should protect workers by enforcing safety rules.” As with American parents, almost all (97%) of Ontario parents indicated their beliefs that it is important or very important to have laws …” limiting the kinds of tasks teenagers are allowed to do” while 98% of Ontario parents agreed that it was important or very important to limit “… the kinds of equipment teens are allowed to use”.

## Discussion

This is one of the first studies to examine the perspectives of parents of teen workers in Canada and builds on earlier work done in the US. As such, it provides new insights into parental views about the work environments and policies affecting their children. We cannot generalize to all of Canada from these results. In fact, earlier work discovered that the rates of injury to young workers in Ontario were lower than in other provinces, especially Saskatchewan, suggesting the need for careful review of parental roles in other work settings and different types of industries [12].

Responses of parents of 14–17-year-old workers in Ontario, compared with those of parents of 14–17-year-old US workers, suggest that higher proportions of Ontario parents expressed concerns about working conditions than U.S. parents on almost every issue about which they were queried [8].

Most parents in both the U.S. and Ontario didn’t believe that their adolescents’ work is hazardous. However, the majority of Ontario parents and much lower proportions of US parents expressed concerns about multiple specific issues. Many of the concerns indicated by the Canadian parents in our survey were similar to those expressed in the focus groups conducted by Usher et al. [11] Particularly, Ontario parents were concerned about the risk of assault, whether sexual or physical assault, while being at work during a robbery. This is in the context of a Canadian rate in 2008 for the robbery of 97 per 100,000 and sexual assault/rape in 2008 of 63 per 100,000 [13], compared to U.S. rates of 142.2 per 100,000 (robbery) [14] and of 80 per 100,000 (sexual assault) in 2003 when we collected the US data [15].

In both countries, parents were supportive of governmental regulation of the teen work environment while also assuming roles as parents in helping teens be aware of safety issues. There seems to be a slight gender disparity for these safety concerns, perhaps signaling a perception that males are more involved in hazardous work than females, which would require additional training or regulation. The majority of both Ontario and US parents wish for youth to finish work by 9 PM and 10 PM for those under 16 years old and 16–17 years old, respectively.

Because our parent survey was coupled with a survey of working adolescents, we sampled families where teens and parents lived together. Parents whose children live independently likely have different views or ways of interacting with their children around worker safety issues.

There is a great opportunity for parents and their working children to stand together for occupational safety through ensuring that workplace safety policies are enacted and enforced. Grant-Smith and McDonald found instances of youth safety ‘self-advocacy’ in their study of Australian youth workers, the opportunity of a young person feeling empowered to recognize and call-out workplace hazards [16]. Parents are ideal facilitators of developing such knowledge and skills in their children. Recent work in Canada has shown that parent’s communication about expectations for safe work behavior is associated with reducing risk-taking by young workers [17]. Engagement of parents with young workers may require a level of vigilance and active involvement on the part of parents as adolescents often actively seek to increase their privacy and autonomy and in which parent-child communication may prove more challenging [18].

## Conclusions

This is one of the first studies to examine parent attitudes of adolescent workplace safety outside the U.S. These results suggest that Ontario parents are concerned about the safety of their working teens and likely can be important allies with safety professionals and policymakers in promoting safer work environments for adolescents. Despite the possibilities of successfully engaging parents of working youth as part of addressing worker’s safety, the primary responsibility for worker safety lies with the employers and policymakers. Some of these issues are articulated in a series of joint US-Canadian series of meetings that developed a set of both research and policy recommendations to move the field forward [19].

## Supplementary Information

**Additional file 1: Table S1.** Ontario parents’ involvement in teen work by gender of parent and teen worker, 2008

**Additional file 2: Table S2.** Ontario parents’ (*n*=507) concerns about adolescent work by a gender of parent and teen worker, 2008

**Additional file 3: Table S3.** Beliefs of Ontario parents about teen work hours when the working teens are attending school, stratified by gender of parent and teen worker, 2008 (n = 507)

**Additional file 4.**

## Data Availability

The datasets generated during and/or analyzed during the current study are not publicly available. Questions about access can be directed to the Study PI ‘s (Drs. Runyan and Lewko).

## Abbreviations

REB: Research Ethics Board
ORE: Office of Research Ethics
CMA: Census Metropolitan Area
U.S.: United States
