# Supplementary material for: Perspectives of parents of working adolescents in Ontario, Canada
Source: BMC Public Health. 2021 Feb 9;21:323. doi: 10.1186/s12889-021-10377-9 (PMC7871646; doi:10.1186/s12889-021-10377-9)
Supplement: Supplementary file 1 — Additional file 1: Table S1. Ontario parents’ involvement in teen work by gender of parent and teen worker, 2008 [file 12889_2021_10377_MOESM1_ESM.docx]

**Supplemental Table 1: Ontario parents’ involvement in teen work by gender of parent and teen worker, 2008**

| **Parent** | **Father (n=111)** | | **Mother (n=396)** | | **Father** | **Mother** |
| --- | --- | --- | --- | --- | --- | --- |
| **Teen Gender** | **Male teen (n=52)**  **% (95% CI)** | **Female teen (n=59)**  **% (95% CI)** | **Male teen (n=192)**  **% (95% CI)** | **Female teen (n=204)**  **% (95% CI)** | **Total teens (n=507)**  **% (95% CI)** | |
| Consider questions to ask about work hours? | 92.2 (80.2, 97.2) | 87.6 (74.7, 94.4) | 89.3 (82.7, 93.6) | 92.5 (87.9, 95.4) | 89.6 (81.6, 94.4) | 90.9 (87.2, 93.7) |
| Identify job opportunities | 88.4 (73.6, 95.4) | 89.6 (78.3, 95.4) | 89.1 (82.5, 93.3) | 89.1 (80.5, 94.2) | 89.0 (80.7, 94.0) | 89.1 (83.9, 92.7) |
| Fill out job applications | 87.1 (73.3, 94.3) | 85.8 (71.8, 93.5) | 86.6 (79.3, 91.7) | 75.7 (65.8, 83.5) | 86.4 (77.3, 92.2) | 80.9 (74.8, 85.9) |
| Prepare for a job interview | 73.8 (58.2, 85.1) | 86.0 (72.3, 93.5) | 86.1 (78.3, 91.4) | 73.5 (63.1, 81.8) | 80.4 (70.3, 87.7) | 79.6 (73.0, 84.4) |
| Consider questions to ask about job tasks | 78.4 (63.6, 88.3) | 78.2 (63.1, 88.2) | 79.8 (72.3, 85.6) | 76.6 (66.6, 84.3) | 78.3 (68.1, 85.8) | 78.1 (71.9, 83.2) |
| Met direct supervisor | 73.7 (59.0, 84.5) | 74.1 (59.2, 84.9) | 67.7 (58.3, 75.8) | 70.1 (61.1, 77.7) | 73.9 (63.7, 82.0) | 68.9 (62.4, 74.7) |
| Handle difficult issues other than about safety | 70.3 (54.8, 82.2) | 48.2 (31.7, 65.1) | 63.3 (54.4, 71.3) | 72.5 (63.0, 80.3) | 58.3 (46.0, 69.7) | 68.1 (61.7, 73.8) |
| Helped teen get more training to do a job | 67.0 (52.0, 79.2) | 56.1 (39.4, 71.5) | 63.2 (54.7, 70.9) | 64.9 (56.5, 72.5) | 61.1 (49.8, 71.2) | 64.1 (58.3, 69.5) |
| Visited workplace to monitor conditions | 64.1 (49.1, 76.7) | 60.4 (41.3, 76.8) | 57.4 (48.4, 65.9) | 69.3 (59.8, 77.4) | 62.1 (49.5, 73.2) | 63.5 (57.0, 69.6) |
| Helped teen learn about worker’s rights | 51.2 (36.9, 66.3) | 41.4 (26.3, 58.3) | 59.4 (50.7, 67.6) | 66.0 (56.9, 74.1) | 46.1 (34.9, 57.8) | 62.8 (56.6, 68.6) |
| Consider questions to ask about workplace safety | 49.9 (35.2, 64.7) | 59.3 (42.9, 73.9) | 53.3(44.5, 61.9) | 63.5 (54.3, 71.9) | 55.0 (43.5, 65.9) | 58.6 (52.3, 64.7) |
| Encouraged teen to report a violation about teen’s work to a government agency? | 21.0 (10.4, 37.9) | 18.5 (8.4, 35.8) | 16.7 (10.9, 24.7) | 19.6 (12.5, 29.3) | 19.6 (11.7, 31.1) | 18.2 (13.3, 24.3) |
| Encouraged teen to quit a job because you were concerned about teen getting injured on the job? | 19.5 (9.5, 35.9) | 2.7 (1.0, 11.0) | 15.8 (10.1, 23.9) | 14.9 (8.8, 24.3) | 10.4 (5.2, 19.4) | 15.3 (10.9, 21.1) |
