# Supplementary material for: Perspectives of parents of working adolescents in Ontario, Canada
Source: BMC Public Health. 2021 Feb 9;21:323. doi: 10.1186/s12889-021-10377-9 (PMC7871646; doi:10.1186/s12889-021-10377-9)
Supplement: Supplementary file 2 — Additional file 2: Table S2. Ontario parents’ (n=507) concerns about adolescent work by a gender of parent and teen worker, 2008 [file 12889_2021_10377_MOESM2_ESM.docx]

**Supplemental Table 2. Ontario parents’ (n=507) concerns about adolescent work by a gender of parent and teen worker, 2008**

|  |  | | | | | |
| --- | --- | --- | --- | --- | --- | --- |
| **Parents indicating ‘‘very concerned’’ or ‘‘somewhat concerned’’** | **Father (n=111)** | | **Mother (n=396)** | | **Father (n=111)** | **Mother (n=396)** |
|  | **Male teen**  **(n=52)**  **% (95% CI)** | **Female teen**  **(n=59)**  **% (95% CI)** | **Male teen**  **(n=192)**  **% (95% CI)** | **Female teen**  **(n=204)**  **% (95% CI)** | **Total – all teens**  **(n=507)**  **%(95% CI)** | |
| Not using protective equipment or clothing | 61.8 (47.0, 74.8) | 36.8 (20.6, 56.6) | 61.0 (52.3, 69.1) | 40.5 (31.7, 50.0) | 48.3 (36.7, 59.9) | 50.4 (43.9, 56.9) |
| Working late at night | 56.7 (41.7, 70.5) | 58.5 (41.7, 73.6) | 56.9 (48.1,65.4 ) | 56.6 (47.2, 65.6) | 57.7 (46.2, 68.4) | 56.8 (50.3, 62.9) |
| Not having safety training to do and complete job tasks safely | 72.5 (57.8, 83.5) | 50.6 (33.8, 67.2) | 64.0 (55.5, 71.8) | 49.8 (40.3, 59.2) | 60.5 (49.1, 71.0) | 56.6 (50.2, 62.9) |
| Not having safety training to identify risky & hazardous conditions on the job | 68.9 (54.2, 80.5) | 55.3 (38.5, 70.9) | 61.4 (52.4, 69.6) | 54.3 (44.8, 63.6) | 61.5 (50.1, 71.7) | 57.7 (51.2, 64.0) |
| Not having received adequate training. | 60.2 (45.3, 73.4) | 52.0 (35.3, 68.3) | 52.2 (43.4, 60.9) | 50.2 (40.8, 59.6) | 55.8 (44.3, 66.6) | 51.1 (44.7, 57.6) |
| Not getting enough sleep because of his/her job | 54.4 (39.5, 68.5) | 62.2 (45.3, 76.6) | 58.3 (49.5, 66.7) | 56.1 (46.6, 65.3) | 58.6 (47.2, 69.2) | 57.2 (50.6, 63.5) |
| Getting behind in school work because of his/her job | 71.2 (56.4, 82.6) | 70.4 (55.2, 82.1) | 69.7 (61.1, 77.1) | 68.1 (60.1, 75.2) | 70.8 (60.4, 79.4) | 68.9 (63.2, 74.1) |
| Being rushed on the job. | 64.6 (49.7, 77.1) | 62.1 (45.3, 76.4) | 64.9 (56.3, 72.6) | 54.2 (44.7, 63.4) | 63.2 (51.9, 73.2) | 59.2 (52.9, 65.5) |
| Handling hazardous equipment, chemicals or toxic substances. | 59.8 (44.9, 73.0) | 42.4 (25.9, 60.8) | 53.6 (44.8, 62.2) | 40.5 (31.5, 50.3) | 50.4 (38.9, 61.9) | 46.8 (40.4, 53.3) |
| Doing hazardous tasks. | 75.2 (61.0, 85.6) | 50.5 (33.8, 67.2) | 63.7 (54.9, 71.7) | 51.7 (42.5, 60.8) | 61.9 (50.6, 71.9) | 57.5 (51.1, 63.6) |
| Working alone. | 55.0 (40.1, 69.0) | 57.7 (41.0, 72.8) | 53.6 (44.7, 62.2) | 48.2 (38.8, 57.7) | 56.5 (45.1, 67.2) | 50.8 (44.2, 57.3) |
| Getting physically or sexually assaulted. | 48.1 (33.5, 63.1) | 68.9 (52.1, 81.8) | 52.1 (43.3, 60.8) | 63.4 (54.2, 71.8) | 59.4 (47.9, 69.9) | 57.9 (51.6, 64.1) |
| Being at work during a robbery. | 68.5 (53.8, 80.2) | 85.2 (72.2, 92.7) | 69.0 (60.3, 76.6) | 78.2 (69.8, 84.8) | 77.6 (67.9, 84.9) | 73.7 (67.9, 78.9) |
