# Supplementary material for: Perspectives of parents of working adolescents in Ontario, Canada
Source: BMC Public Health. 2021 Feb 9;21:323. doi: 10.1186/s12889-021-10377-9 (PMC7871646; doi:10.1186/s12889-021-10377-9)
Supplement: Supplementary file 3 — Additional file 3: Table S3. Beliefs of Ontario parents about teen work hours when the working teens are attending school, stratified by gender of parent and teen worker, 2008 (n = 507) [file 12889_2021_10377_MOESM3_ESM.docx]

**Supplemental Table 3. Beliefs of Ontario parents about teen work hours when the working teens are attending school, stratified by gender of parent and teen worker, 2008 (n= 507)**

| **Parent** | **Father (n=111)** | | **Mother (n=396)** | | | **Father (n=111)** | **Mother (n=396)** |
| --- | --- | --- | --- | --- | --- | --- | --- |
| **Teen Gender** | **Male teen**  **% (95% CIs)** | **Female teen**  **% (95% CIs)** | **Male teen**  **% (95% CIs)** | **Female teen**  **% (95% CIs)** | **Total teens (n=507)**  **% (95% CIs)** | | |
| **In your opinion, what is the latest hour that a teen worker UNDER 16 should be allowed to work when there is school the next day?** | | | | | | | |
| Earlier than 8PM | 10.7 (4.7, 22.4) | 25.2 (10.7, 48.7) | 15.2 (9.8, 22.9) | 16.8 (10.9, 25.2) | 18.6(9.4, 33.4) | | 16.1 (11.9, 21.4) |
| 8PM | 28.6 (16.8, 44.4) | 10.9 (4.8, 22.8) | 24.8 (17.1,34.5) | 21.3 (14.6, 30.0) | 18.9 (11.9, 28.8) | | 23.0 (17.7, 29.3) |
| 9PM | 46.4 (32.0, 61.4) | 43.7 (28.0, 60.7) | 39.8 (31.8, 48.5) | 43.6 (34.6, 53.2) | 44.9 (33.9, 56.5) | | 41.8 (35.5, 48.3) |
| 10PM | 13.1 (5.9, 26.6) | 17.5 (9.0, 31.3) | 14.0 (9.6, 20.1) | 14.2 (8.6, 22.3) | 15.5 (9.4, 24.5) | | 14.1 (10.3, 19.0) |
| 11PM | 1.3 (0.2, 8.5) | 1.8 (0.2, 11.6) | 2.6(1.1, 6.0) | 1.5 (1.0, 4.3) | 1.5 (0.4, 6.2) | | 2.0 (1.1, 3.9) |
| 12AM or later | - | - | 1.0 (0.2, 3.1) | 0.4 (0.1, 3.1) | - | | 1.0 (0.1, 1.8) |
| Refused or Don’t Know | - | 1.0 (0.1, 6.4) | 2.8 (1.0, 7.2) | 2.1 (1.0, 6.5) | 1.0 (0.1, 3.5) | | 2.4 (1.1, 5.1) |
| **In your opinion, what is the latest hour that a 16 - 17 year old should be allowed to work when there is school the next day?** | | | | | | | |
| Earlier than 8PM | 5.2 (1.6, 15.7) | 14.6 (3.2, 46.7) | 2.4 (1.0, 5.9) | 5.7 (2.1, 14.8) | 10.3 (3.1, 29.3 ) | | 4.1 (1.9, 8.7) |
| 8PM | 9.2 (3.0, 25.0) | 3.7 (1.1, 11.8) | 5.4 (2.6, 10.7) | 9.1 (4.3, 18.4) | 6.2 (2.6, 14.0) | | 7.3 (4.2, 12.4) |
| 9PM | 37.5 (24.1, 53.2) | 34.5 (20.8, 51.4) | 40.9 (32.3, 50.0) | 31.7 (24.1, 40.5) | 35.9 (25.7, 47.5) | | 36.1 (30.1, 42.6) |
| 10PM | 36.8 (24.0, 51.7) | 30.9 (18.5, 46.7) | 35.0 (27.3, 43.7) | 40.2 (31.1, 49.9) | 33.6 (24.2, 44.5) | | 37.7 (31.6. 44.2) |
| 11PM | 7.2 (2.8, 17.1) | 13.7 (6.7, 25.8) | 13.6 (8.9, 20.2) | 10.0 (6.5, 15.1) | 10.7 (6.1, 18.1) | | 11.7 (8.7, 15.7) |
| 12AM or later | 4.0 (1.0, 23.8) | 1.0 (0.1, 6.4) | 1.6 (1.0, 4.2) | 2.4 (1.0, 5.5) | 2.4 (1.0, 10.9) | | 2.0 (1.1, 3.8) |
| Refused or Don’t Know | - | 1.8 (0.4, 6.9) | 1.0 (0.1, 6.5) | 1.0 (0.2, 3.6) | 1.0 (0.2, 3.8) | | 1.0 (0.3, 3.0) |
