# Supplementary material for: Perspectives of parents of working adolescents in Ontario, Canada
Source: BMC Public Health. 2021 Feb 9;21:323. doi: 10.1186/s12889-021-10377-9 (PMC7871646; doi:10.1186/s12889-021-10377-9)
Supplement: Supplementary file 4 — Additional file 4. [file 12889_2021_10377_MOESM4_ESM.docx]

| **No.** | **Question and Response Set** | **Comments** |
| --- | --- | --- |
| P1 | Is [NAME OF CHILD] working at [REFERENT JOB] now?  1= YES---P3  2= NO---P2  3= REFUSED---P3  4= DON’T KNOW---P3 |  |
| P2 | Why has [NAME OF CHILD] stopped working at [REFERENT JOB]?  1= RETURNED TO SCHOOL  2= JOB ENDED/SEASONAL  3= THEY WERE FIRED OR LAID OFF  4= THEY GOT A BETTER/DIFFERENT JOB  5= PARENT MADE THEM QUIT  6= TEEN DIDN’T LIKE THE JOB  7= GOT HURT  8= JOB TOO HARD  9= HOURS WERE WRONG (TOO LONG, TOO SHORT, TOO EARLY, TOO LATE)  10= OTHER REASON (Please specify: P2OTH__)  11= REFUSED  12= DON’T KNOW | - Do not read codes. - Code appropriately given the Parent’s verbatim response - If unsure, then code 10 (OTHER) and enter the parent’s response. |
| P3 | Why do you think [NAME OF CHILD]  Works?  1 =SPENDING MONEY  2 =SAVING MONEY  3 =EXPERIENCE  4 =FAMILY BUSINESS  5 =PARENTS WANT HIM/HER TO WORK  6 =OTHER REASON (PLEASE SPECIFY)  9 =DON'T KNOW |  |
| P4 | Has [NAME OF CHILD] ever been in a supervisor position at work?  1=YES---Go to next  2=NO ---P5  3=REFUSED ---P5  4=DON’T KNOW | A supervisor (team leader; crew leader; manager) is someone who has been designated by management to be a supervisor. |
| P4a | What kind of training did [NAME OF CHILD] receive to manage young workers?  1=NONE  2= MANAGEMENT COURSE  3= MENTORING BY MANAGER  4= INFORMED OF LEGAL SUPERVISOR RESPONSIBILITIES  5= WRITTEN INSTRUCTIONS  6= INSTRUCTIONAL VIDEOS  7= OTHER (Please Specify: T ##OTH )---  8= REFUSED | Please check off all that apply |
| P5 | Now I have several questions about the work that [NAME OF CHILD] did/does while working at [REFERENT JOB].  During the last two months, while working at [REFERENT JOB], has [NAME OF CHILD] worked on a night before a school day?  1= YES---P5a  2= NO---P6  3= REFUSED---P6  4= DON’T KNOW---P6 |  |
| P5a | During the last two months about how many nights each week, on average, has [NAME OF CHILD] worked past 7 pm at [REFERENT JOB] on a night before a school day?  __ (Open numerical format 1-7)  6= REFUSED  7 = DON’T KNOW |  |
| P5b | Has [NAME OF CHILD] worked past 9 pm at [REFERENT JOB] on a night before a school day?  1= YES---P5C.  2= NO---P6  3= REFUSED---P6  4= DON’T KNOW---P6 |  |
| P5c | During the last two months, about how many nights each week, on average, has [NAME OF CHILD] worked past 9 pm at [REFERENT JOB] on a night before a school day?  __ (Open numerical format 1-7)  6= REFUSED  7 = DON’T KNOW |  |
| P5d | Has [NAME OF CHILD] worked past 11 pm at [REFERENT JOB] on a night before a school day?  1= YES---P5E  2= NO---P6  3= REFUSED---P6  4= DON’T KNOW---P6 |  |
| P5e | During the last two months, about how many nights each week, on average, has [NAME OF CHILD] worked past 11 pm at [REFERENT JOB] on a night before a school day?  __ (Open numerical format 1-7)  6= REFUSED  7 = DON’T KNOW |  |
| P6 | While [NAME OF CHILD] has been working at [REFERENT JOB], have you ever visited [HIM/HER] at the work place to check on or monitor the working conditions?  1= YES---P5A.  2= NO---P6  3= REFUSED---P6  4= DON’T KNOW---P6 |  |
| P6a | While [NAME OF CHILD] has been working at [REFERENT JOB], how many times in the last two months have you visited [HIM/HER] at the work place?  __ DAYS (Open numerical format 0-99)  88= REFUSED  99= DON’T KNOW |  |
| P7 | Have you ever met [NAME OF CHILD]’s direct supervisor at [REFERENT JOB]?  1= YES  2= NO  3= REFUSED  4= DON’T KNOW |  |
| P8 | In a typical work week while working at [NAME OF CHILD]’s [REFERENT JOB], how many days does (did) he/she work some or part of the day without a qualified supervisor available at the worksite?  ___ DAYS PER WEEK (OPEN NUMERICAL FORMAT)  8= REFUSED  9 = DON’T KNOW |  |
| P9 | During a typical work week at [NAME OF CHILD]’s job, how many days per week is he/she the only person at the worksite during daylight hours?  ___ DAYS PER WEEK (OPEN NUMERICAL FORMAT <10)  8= REFUSED  9 = DON’T KNOW |  |
| P10 | During a typical work week at [NAME OF CHILD]’s job, how many nights is he/she the only worker at the worksite after dark for at least half an hour?  ___ NIGHTS PER WEEK  (OPEN NUMERICAL FORMAT, <10)  8= REFUSED  9 = DON’T KNOW |  |
| P11 | In general, how familiar are you with any problems or difficulties [NAME OF CHILD] has/had at [REFERENT JOB]?  Would you say that you are…  1= VERY FAMILIAR  2= SOMEWHAT FAMILIAR  3= SLIGHTLY FAMILIAR  4= NOT AT ALL FAMILIAR  5= REFUSED  6= DON’T KNOW |  |
| P11a | How involved have you been in giving [NAME OF CHILD] advice about tasks [HE/SHE] performs at [REFERENT JOB]. Would you say you have been…  1=VERY INVOLVED  2=SOMEWHAT INVOLVED  3=NOT AT ALL INVOLVED  4=REFUSED  5=DON’T KNOW |  |
| P12a | Now I’m going to ask you about tasks that [NAME OF CHILD] may have done while working at [REFERENT JOB]. If these tasks do not apply, simply answer “no”. If you don’t know or aren’t sure, just tell me so.  While working at [REFERENT JOB] has [NAME OF CHILD]:  Handled cash?  1= YES  2= NO  3= REFUSED  4= DON’T KNOW |  |
| P12b | While working at [REFERENT JOB] has [NAME OF CHILD]:  Used sharp knives?  1= YES  2= NO  3= REFUSED  4= DON’T KNOW |  |
| P12c | While working at [REFERENT JOB] has [NAME OF CHILD]:  Used a power slicing machine or grinder?  1= YES  2= NO  3= REFUSED  4= DON’T KNOW |  |
| P12d | While working at [REFERENT JOB] has [NAME OF CHILD]:  Done cleaning tasks, including mopping, scrubbing, sweeping, or taking out trash?  1= YES  2= NO  3= REFUSED  4= DON’T KNOW |  |
| P12e | While working at [REFERENT JOB] has [NAME OF CHILD]:  Driven a car or truck while working at [REFERENT JOB]?  1= YES  2= NO  3= REFUSED  4= DON’T KNOW | Note to interviewer that this does not include driving to and from work. |
| P12f | While working at [REFERENT JOB] has [NAME OF CHILD]:  Worked with chemicals that you thought were dangerous?  1= YES  2= NO  3= REFUSED  4= DON’T KNOW |  |
| P12g | While working at [REFERENT JOB] has [NAME OF CHILD]:  Worked on ladders, roofs, or scaffolding higher than 6 feet ?  1= YES---P9h  2= NO ---P9i  3= REFUSED---P9i  4= DON’T KNOW---P9i |  |
| P12h | While working at [REFERENT JOB] has [NAME OF CHILD]:  Was equipment like a harness, railing or wall, used to prevent falls?  1= YES  2= NO  3= REFUSED  4= DON’T KNOW |  |
| P12i | While working at [REFERENT JOB] has [NAME OF CHILD]:  Operated heavy equipment or machinery (e.g. forklifts, tractors)?  1= YES  2= NO  3= REFUSED  4= DON’T KNOW |  |
| P12j | While working at [REFERENT JOB] has [NAME OF CHILD]:  Moved or lifted heavy objects – by that I mean objects that weigh 50 pounds (23 kilograms) or more…”  1= YES  2= NO  3= REFUSED  4= DON’T KNOW | - |
| P12k | While working at [REFERENT JOB] has [NAME OF CHILD]:  Moved or lifted heavy objects with the help of others or devices such as carts, lifts, etc?  1= YES  2= NO  3= REFUSED  4= DON’T KNOW |  |
| P13 | Do you consider any of [NAME OF CHILD]’s job tasks at [REFERENT JOB] to be hazardous?  1= YES  2= NO  3= REFUSED  4= DON’T KNOW | “Hazardous” as defined by the parent. |
| P13a | Which job tasks do you consider hazardous?  1= MOVING HEAVY OBJECTS  2= USING SHARP CUTTING INSTRUMENTS SUCH AS CASE CUTTERS, KNIVES, ETC  3= HOT LIQUIDS AND GREASE  4= HOT SURFACES (IE OVENS, GRILLS, ETC)  5= POWERED MACHINERY  6= EXPOSURE TO NEEDLES, BLOOD PRODUCTS, OR MEDICAL WASTE  7= SLIPPERY FLOORS  8= FALLING OBJECTS  9= LOUD NOISES  10=WHERE HEAVY EQUIPMENT IS OPERATING  11= CLUTTERED AND CROWDED WORKPLACE  12= WORKING ALONE  13= WORKING WITHOUT ADEQUATE SUPERVISION  14=WORKING WITHOUT ADEQUATE TRAINING  15= OTHER (Please Specify: T14.aOTH )  16=REFUSED  17=DON’T KNOW | Comment:  Select all that apply |
| P14a | Now I’m going to list some things that some parents of working teens might worry about. I would like you to tell me how concerned you are about each when you think of [NAME OF CHILD] working at [REFERENT JOB].  Not using protective equipment or clothing. Are you …  1= VERY CONCERNED  2= SOMEWHAT CONCERNED  3= NOT AT ALL CONCERNED  4= REFUSED  5= DON’T KNOW | Questions 14a – 14n address concern about perceived control to perform safe work practices.  All 14 questions use a 3-point Likert scale (very concerned, somewhat concerned, and not at all concerned). |
| P14b | Doing hazardous tasks  1= VERY CONCERNED  2= SOMEWHAT CONCERNED  3= NOT AT ALL CONCERNED  4= REFUSED  5= DON’T KNOW |  |
| P14c | Handling hazardous equipment, chemicals or toxic substances.  1= VERY CONCERNED  2= SOMEWHAT CONCERNED  3= NOT AT ALL CONCERNED  4= REFUSED  5= DON’T KNOW |  |
| P14d | Not having safety training and complete job tasks safely.  1= VERY CONCERNED  2= SOMEWHAT CONCERNED  3= NOT AT ALL CONCERNED  4= REFUSED  5= DON’T KNOW |  |
| P14e | Not having safety training to identify risky and hazardous conditions on the job.  1= VERY CONCERNED  2= SOMEWHAT CONCERNED  3= NOT AT ALL CONCERNED  4= REFUSED  5= DON’T KNOW |  |
| P14f | Not having safety training about worker rights and responsibilities at work.  1= VERY CONCERNED  2= SOMEWHAT CONCERNED  3= NOT AT ALL CONCERNED  4= REFUSED  5= DON’T KNOW |  |
| P14g | Working alone  1= VERY CONCERNED  2= SOMEWHAT CONCERNED  3= NOT AT ALL CONCERNED  4= REFUSED  5= DON’T KNOW |  |
| P14h | Working too late at night  1= VERY CONCERNED  2= SOMEWHAT CONCERNED  3= NOT AT ALL CONCERNED  4= REFUSED  5= DON’T KNOW |  |
| P14i | Getting physically or sexually assaulted  1= VERY CONCERNED  2= SOMEWHAT CONCERNED  3= NOT AT ALL CONCERNED  4= REFUSED  5= DON’T KNOW |  |
| P14j | Being there during a robbery  1= VERY CONCERNED  2= SOMEWHAT CONCERNED  3= NOT AT ALL CONCERNED  4= REFUSED  5= DON’T KNOW |  |
| P14k | Getting behind in school work because of his/her job  1= VERY CONCERNED  2= SOMEWHAT CONCERNED  3= NOT AT ALL CONCERNED  4= REFUSED  5= DON’T KNOW |  |
| P14l | Being rushed on the job  1= VERY CONCERNED  2= SOMEWHAT CONCERNED  3= NOT AT ALL CONCERNED  4= REFUSED  5= DON’T KNOW |  |
| P14m | Not getting enough sleep because of his/her job  1= VERY CONCERNED  2= SOMEWHAT CONCERNED  3= NOT AT ALL CONCERNED  4= REFUSED  5= DON’T KNOW |  |
| P14n | Not having received adequate training.  1= VERY CONCERNED  2= SOMEWHAT CONCERNED  3= NOT AT ALL CONCERNED  4= REFUSED  5= DON’T KNOW |  |
| P15a | Now I have some general questions about how important you think certain things are in preventing any teenager from being injured on-the-job.  How important is it…  …that teens get on-the-job training on how to perform basic job tasks?  1= VERY IMPORTANT  2= SOMEWHAT IMPORTANT  3= SLIGHTLY IMPORTANT  4= NOT IMPORTANT AT ALL  5= REFUSED  6= DON’T KNOW |  |
| P15b | …that teens have safety equipment or safety clothing?  1= VERY IMPORTANT  2= SOMEWHAT IMPORTANT  3= SLIGHTLY IMPORTANT  4= NOT IMPORTANT AT ALL  5= REFUSED  6= DON’T KNOW |  |
| P15c | …that teens have a qualified adult supervisor on the job?  1= VERY IMPORTANT  2= SOMEWHAT IMPORTANT  3= SLIGHTLY IMPORTANT  4= NOT IMPORTANT AT ALL  5= REFUSED  6= DON’T KNOW | Adult refers to someone over the age of 25.  Comparability issue, Runyan- US study Adult =21 |
| P15d | …that teens avoid peer pressure to act in certain ways?  1= VERY IMPORTANT  2= SOMEWHAT IMPORTANT  3= SLIGHTLY IMPORTANT  4= NOT IMPORTANT AT ALL  5= REFUSED  6= DON’T KNOW |  |
| P15e | …that teens ask lots of questions about tasks and rules?  1= VERY IMPORTANT  2= SOMEWHAT IMPORTANT  3= SLIGHTLY IMPORTANT  4= NOT IMPORTANT AT ALL  5= REFUSED  6= DON’T KNOW |  |
| P15f | …that teens have parents or guardians who help them look out for safety issues?  1= VERY IMPORTANT  2= SOMEWHAT IMPORTANT  3= SLIGHTLY IMPORTANT  4= NOT IMPORTANT AT ALL  5= REFUSED  6= DON’T KNOW |  |
| P15g | …that there are laws limiting the kinds of tasks teenagers are allowed to do?  1= VERY IMPORTANT  2= SOMEWHAT IMPORTANT  3= SLIGHTLY IMPORTANT  4= NOT IMPORTANT AT ALL  5= REFUSED  6= DON’T KNOW |  |
| P15h | …that there are laws limiting the kinds of equipment teenagers are allowed to use?  1= VERY IMPORTANT  2= SOMEWHAT IMPORTANT  3= SLIGHTLY IMPORTANT  4= NOT IMPORTANT AT ALL  5= REFUSED  6= DON’T KNOW |  |
| P16 | In your opinion, what is the maximum number of hours a teen worker under age 18 and still in school should be allowed to work during a week when school is in session?  __ (OPEN NUMERICAL FORMAT, <99)  _88=REFUSED  _99=DON’T KNOW |  |
| P17 | In your opinion, what is the latest hour that a 16 or 17 year old should be allowed to work when there is school the next day?  1= EARLIER THAN 8 PM  2= 8 PM  3= 9 PM  4= 10 PM  5= 11 PM  6= 12 MIDNIGHT  7= LATER THAN 12 MIDNIGHT, BEFORE 2 AM  8= NO TIME RESTRICTION AT ALL  9= REFUSED  10 = DON’T KNOW. | Do not read responses unless necessary to probe |
| P18 | In your opinion, what is the latest hour that a teen worker under 16 should be allowed to work when there is school the next day?  1=EARLIER THAN 8 PM  2=8 PM  3=9 PM  4=10 PM  5=11 PM  6=12 MIDNIGHT  7= LATER THAN 12 MIDNIGHT, BEFORE 2 AM  8= NO TIME RESTRICTION AT ALL  9= REFUSED  10 = DON’T KNOW | - Do not read responses unless necessary to probe. - Emphasize under 16 |
| P19a | Thinking again about the work [NAME OF CHILD] does at [REFERENT JOB], how strongly do you agree or disagree with each of the following statements about that job.  I don’t want [NAME OF CHILD] to work as many hours as [HE/SHE] does. Do you…  1= STRONGLY AGREE  2= SOMEWHAT AGREE  3= SOMEWHAT DISAGREE  4= STRONGLY DISAGREE  5= REFUSED  6= DON’T KNOW | - Questions P19a – P19d address beliefs about safe work practices. - These 4 questions use a 4-point Likert scale (e.g. strongly agree, somewhat agree, somewhat disagree, and strongly disagree). |
| P19b | I am concerned that working at [REFERENT JOB] could be dangerous for [NAME OF CHILD]?  1= STRONGLY AGREE  2= SOMEWHAT AGREE  3= SOMEWHAT DISAGREE  4= STRONGLY DISAGREE  5= REFUSED  6= DON’T KNOW |  |
| P19c | I am confident that my teenager knows how to keep [HIM/HER] self safe while on the job.  1= STRONGLY AGREE  2= SOMEWHAT AGREE  3= SOMEWHAT DISAGREE  4= STRONGLY DISAGREE  5= REFUSED  6= DON’T KNOW |  |
| P19d | I am confident that my teenager knows [HIS/HER] rights to refuse unsafe work.  1= STRONGLY AGREE  2= SOMEWHAT AGREE  3= SOMEWHAT DISAGREE  4= STRONGLY DISAGREE  5= REFUSED  6= DON’T KNOW |  |
| P20a | Now, I’m going to read some general statements about teens and work. Please tell me if you strongly agree, somewhat agree, somewhat disagree, or strongly disagree with each statement.  Accidents at work just happen and there is little that teen employees can do to avoid injuries.  1=STRONGLY AGREE  2=SOMEWHAT AGREE  3=SOMEWHAT DISAGREE  4=STRONGLY DISAGREE  5=REFUSED  6=DON’T KNOW |  |
| P20b | Laws that keep teenagers from working late at night on school nights are a bad idea.  1=STRONGLY AGREE  2=SOMEWHAT AGREE  3=SOMEWHAT DISAGREE  4=STRONGLY DISAGREE  5=REFUSED  6=DON’T KNOW |  |
| P20c | Laws should limit the number of daily and weekly hours that teenagers can work.  1=STRONGLY AGREE  2=SOMEWHAT AGREE  3=SOMEWHAT DISAGREE  4=STRONGLY DISAGREE  5=REFUSED  6=DON’T KNOW |  |
| P20d | Parents, not laws, should decide what kinds of work their teenagers can do.  1= STRONGLY AGREE  2= SOMEWHAT AGREE  3= SOMEWHAT DISAGREE  4= STRONGLY DISAGREE  5= REFUSED  6= DON’T KNOW |  |
| P20e | Employers should protect workers by enforcing safety rules.  1= STRONGLY AGREE  2= SOMEWHAT AGREE  3= SOMEWHAT DISAGREE  4= STRONGLY DISAGREE  5= REFUSED  6= DON’T KNOW |  |
| P21 | Now I’d like you to think about the possibility that your child might get injured while working at [REFERENT JOB].  How likely is it that [NAME OF CHILD] will be injured at [REFERENT JOB] seriously enough that [HE/SHE] will need medical attention or will miss one or more days of school or work during the next 12 months.  Would you say it is…  1= VERY LIKELY  2= SOMEWHAT LIKELY  3= SOMEWHAT UNLIKELY  4= VERY UNLIKELY  5= REFUSED  6= DON’T KNOW | - Medical attention here means that teen saw a nurse - Or doctor, or visited a clinic, emergency room or hospital. |
| P22a | Parents address their child’s workplace safety concerns in different ways. For the next series of questions, I’m going to read a list of ways some parents address workplace concerns with their children. For each statement, please tell me how likely it is you would do each of the following things.  If you were concerned that the work your teenager was doing might be dangerous, how likely would you be to talk directly to [HIS/HER] supervisor about the problem?  1= VERY LIKELY  2= SOMEWHAT LIKELY  3= SOMEWHAT UNLIKELY  4= VERY UNLIKELY  5= REFUSED  6= DON’T KNOW | - Questions 22a – 22g address dealing with workplace safety practices. - All 7 use a 4-point Likert scale (e.g., very likely, somewhat likely, somewhat unlikely, and very unlikely). |
| P22b | If you thought your child’s work situation was dangerous, how likely would you be to encourage him/her to talk directly to his/her supervisor about the problem?  1= VERY LIKELY  2= SOMEWHAT LIKELY  3= SOMEWHAT UNLIKELY  4= VERY UNLIKELY  5= REFUSED  6= DON’T KNOW |  |
| P22c | How likely would you be to contact the Ministry of Labour (MOL), the Workplace Safety Insurance Board (WSIB) or some other government agency yourself?  1= VERY LIKELY  2= SOMEWHAT LIKELY  3= SOMEWHAT UNLIKELY  4= VERY UNLIKELY  5= REFUSED  6= DON’T KNOW |  |
| P22d | How likely would you be to tell your child to file a complaint WITH WSIB OR Ministry of Labour?  1= VERY LIKELY  2= SOMEWHAT LIKELY  3= SOMEWHAT UNLIKELY  4= VERY UNLIKELY  5= REFUSED  6= DON’T KNOW |  |
| P22e | How likely would you be to wait and see if the problem gets better?  1= VERY LIKELY  2= SOMEWHAT LIKELY  3= SOMEWHAT UNLIKELY  4= VERY UNLIKELY  5= REFUSED  6= DON’T KNOW |  |
| P22f | How likely would you be to tell your teen to get assigned to different duties?  1= VERY LIKELY  2= SOMEWHAT LIKELY  3= SOMEWHAT UNLIKELY  4= VERY UNLIKELY  5= REFUSED  6= DON’T KNOW |  |
| P22g | How likely would you be to tell your teen to quit working for this employer?  1= VERY LIKELY  2= SOMEWHAT LIKELY  3= SOMEWHAT UNLIKELY  4= VERY UNLIKELY  5= REFUSED  6= DON’T KNOW |  |
| P23a | Still thinking about the possibility that your teenager might be in a job that you think is dangerous, how strongly do you agree or disagree with each of these statements about ways you might try to help [HIM/HER]?  I don’t know enough about my teenager’s job to talk to them about safety at work.  1= STRONGLY AGREE  2= SOMEWHAT AGREE  3= SOMEWHAT DISAGREE  4= STRONGLY DISAGREE  5= REFUSED  6= DON’T KNOW |  |
| P23b | My teenager doesn’t want my help.  1= STRONGLY AGREE  2= SOMEWHAT AGREE  3= SOMEWHAT DISAGREE  4= STRONGLY DISAGREE  5= REFUSED  6= DON’T KNOW |  |
| P23c | I think part of teens’ learning is for them to figure out about job safety themselves.  1= STRONGLY AGREE  2= SOMEWHAT AGREE  3= SOMEWHAT DISAGREE  4= STRONGLY DISAGREE  5= REFUSED  6= DON’T KNOW |  |
| P23d | I worry that my teen’s employer will be upset if I try to get involved.  1= STRONGLY AGREE  2= SOMEWHAT AGREE  3= SOMEWHAT DISAGREE  4= STRONGLY DISAGREE  5= REFUSED  6= DON’T KNOW |  |
| P24 | Has [NAME OF CHILD] ever reported a workplace safety problem to you?  1= YES---Q25a  2= NO---Q26a  3= DON’T KNOW---Q26a  4= REFUSAL---Q26a |  |
| P25a | Following the report, did you do any of the following things?  Told [HIM/HER] to talk with [HIS/HER] supervisor or boss?  1= YES  2= NO  3= REFUSED  4= DON’T KNOW |  |
| P25b | Talked directly to a supervisor yourself?  1= YES  2= NO  3= REFUSED  4= DON’T KNOW |  |
| P25c | Visited [HIS/HER] work site to check on the safety issue?  1= YES  2= NO  3= REFUSED  4= DON’T KNOW |  |
| P25d | Reported the safety issue to WSIB. or some other government agency?  1= YES  2= NO  3= REFUSED  4= DON’T KNOW |  |
| P25e | Advised [HIM/HER] to quit working for this employer?  1= YES  2= NO  3= REFUSED  4= DON’T KNOW |  |
| P26a | There are many ways parents might help their children think about decisions related to work?  Have you or another parent/guardian ever helped [HIM/HER]…  Identify job opportunities?  1= YES  2= NO  3= REFUSED  4= DON’T KNOW | - Questions 26a – 26g concern helping teens choose/keep a job. - All 7 are Y/N questions. |
| P26b | Have you or another parent/guardian ever helped [HIM/HER]…  Fill out a job application?  1= YES  2= NO  3= REFUSED  4= DON’T KNOW | It means helped… |
| P26c | Have you or another parent/guardian ever helped [HIM/HER]…  Prepare for a job interview?  1= YES  2= NO  3= REFUSED  4= DON’T KNOW |  |
| P26d | Have you or another parent/guardian ever helped [HIM/HER]…  Consider questions to ask employers about workplace safety?  1= YES  2= NO  3= REFUSED  4= DON’T KNOW |  |
| P26e | Have you or another parent/guardian ever helped [HIM/HER]…  Consider questions to ask about job tasks?  1= YES  2= NO  3= REFUSED  4= DON’T KNOW |  |
| P26f | Have you or another parent/guardian ever helped [HIM/HER]…  Consider questions to ask about work hours?  1= YES  2= NO  3= REFUSED  4= DON’T KNOW |  |
| P26g | Have you or another parent/guardian ever helped [HIM/HER]…  Handle difficult work issues other than about safety?  1= YES  2= NO  4= REFUSED  5= DON’T KNOW |  |
| P27a | Still talking about ways parents might help their children think about decisions related to work.  Have you or another parent or guardian ever…  Encouraged [HIM/HER] to quit a job because you were concerned about [HIM/HER] getting injured on the job?  1= YES  2= NO  3= REFUSED  4= DON’T KNOW |  |
| P27b | Have you or another parent or guardian ever...  Encouraged HIM/HER to learn about worker's rights?  1= YES  2= NO  3= REFUSED  4= DON’T KNOW |  |
| P27c | Have you or another parent or guardian ever...  Encouraged HIM/HER to get more training to do a job?  1= YES  2= NO  3= REFUSED  4= DON’T KNOW |  |
| P27d | Have you or another parent or guardian ever...  Encouraged HIM/HER to report a violation about [NAME OF CHILD] 's work to a government agency?  1= YES  2= NO  3= REFUSED  4= DON’T KNOW |  |
| P28 | While [NAME OF CHILD] has worked at [REFERENT JOB] has [HE/SHE] ever been injured badly enough on the job to miss a day of work or school or to receive medical attention?  1= YES  2= NO  3= REFUSED  4= DON’T KNOW | Interviewer: medical treatment here means seeing a nurse or doctor, or visiting a clinic, emergency room or hospital |
| P28a | While working at previous jobs, was [NAME OF CHILD] ever injured badly enough on the job that [HE/SHE] had to miss a day of work or school or receive medical treatment?  1= YES  2= NO  3= REFUSED  4= DON’T KNOW | Interviewer: medical treatment here means seeing a nurse or doctor, or visiting a clinic, emergency room or hospital |
| P29 | Finally just a couple of background questions about you.  Are you employed?  1= YES---P30  2= NO---P31  3= REFUSED---P31  4= DON’T KNOW---P31 | The last questions collect demographic attributes for stratification. |
| P30 | All together, how many hours of paid work do you do in an average week?  __ (OPEN NURMATICAL FORMAT <1000)  888= REFUSED  999=DON’T KNOW |  |
| P31 | Have you ever been injured badly enough at work that you needed to seek medical attention or miss work for a day or more?  1= YES  2= NO  3= REFUSED  4= DON’T KNOW |  |
| P32 | Now, I have just a few final questions about you. What is your relationship to [NAME OF CHILD]?  01= MOTHER  02= STEPMOTHER  03= FATHER  04= STEPFATHER  05= FEMALE GUARDIAN  06= MALE GUARDIAN  07= GRANDMOTHER  08= GRANDFATHER  09= AUNT  10= UNCLE  11= OLDER BROTHER  12= OLDER SISTER  13= OTHER (Please specify___________)  15= REFUSED  16= DON’T KNOW | Read responses only as necessary to clarify |
| P33 | How old are you?  1=UNDER 30 YEARS OLD  2= 30 TO 39 YEARS OLD  3= 40 TO 49 YEARS OLD  4= 50 TO 59 YEARS OLD  5=60 TO 69 YEARS OLD  6= OVER 70 YEARS OLD  7= REFUSED |  |
| P34 | What's the highest level of education that you have completed thus far?  _ # OF YEARS  _88 REFUSED  _99 DON’T KNOW |  |
| P35 | Who is the head of your household?  1= SELF  2= OTHER  3= REFUSED  4= DON’T KNOW  5= JOINT |  |
| P36 | How would you best describe your racial or ethnic background (CHECK ONLY ONE)? LET RESPONDENT VOLUNTEER RACE.  1= Aboriginal (Inuit, Métis, North American Indian)  2= White (Caucasian)  3= OTHER (SPECIFY: T49OTH )  4=REFUSED  5=DON’T KNOW (PROBE: What’s your race?) | Use silent codes  List generated from Statistics Canada. A more expansive list is available. |
| P37 | What is your total family household income, before taxes?  1= LESS THAN $5000  2= $5000 AND OVER  3= $10,000 AND OVER  4= $15,000 AND OVER  5=$20,000 AND OVER  6= $25,000 AND OVER  7= $30,000 AND OVER  8= $35 000 AND OVER  9= $40,000 AND OVER  10=$45,000 AND OVER  11= $50,000 AND OVER  12=$55 000 AND OVER  13= $60 000 AND OVER  14= $65,000 AND OVER  15= $70,000 AND OVER  16= $75,000 AND OVER  17= $80,000 AND OVER  18= $85,000 AND OVER  19= $90,000 AND OVER  20= $95,000 AND OVER  21= $100,000 AND OVER  22= $150,000 AND OVER  23= $200,000 AND OVER  24= $250,000 AND OVER  25 REFUSED  26 DON'T KNOW |  |
| P38a | Does your household have more than one phone number?  1= YES (GO TO P38b)  2= NO (GO TO P39a)  3= REFUSED (GO TO P39a)  4= DON’T KNOW (GO TO P39a) |  |
| P38b | What are those phone numbers used for? (SELECT ALL THAT APPLY.)  1=CELL PHONE (GO TO P39a)  2=DEDICATED FAX LINE (GO TO P39a)  3=DEDICATED COMPUTER LINE (GOTO P39a)  4=DEDICATED BUSINESS # (GOTO P39a)  5=ADDITIONAL LINES (GO TO P38c)  6=REFUSED (GO TO P39a)  7=DON’T KNOW (GO TO P39a) |  |
| P38c | How many additional lines come to your house?  _ # OF ADDITIONAL LINES (GO TO P38d)  _88 REFUSED (GO TO P39a)  _99 DON’T KNOW (GO TO P39a) |  |
| P38d | So to verify, you have [VALUE OF (P38c) + 1 ] lines that come to your house?  (CELL NUMBERS OR DEDICATED FAX, BUSINESS OR COMPUTER LINES ARE NOT COUNTED HERE.)  1=YES (GOTO P39a)  2=NO (GOTO P38a)  3=REFUSED (GO TO P39a)  4=DON’T KNOW (GO TO P39a) |  |
| P39a | During the past 12 months, has your household been without telephone service for 1 week or more? Please do not include cellular phones in your answer?  1=YES (GO TO P39b)  2=NO (GO TO P40)  3=REFUSED (GO TO P40)  4=DON’T KNOW (GO TO P40) |  |
| P39b | For how long was your household without telephone service in the past 12 months?  __ ENTER NUMBER—IF 1 WEEK OR LESS,ENTER 0 (GO TO P40)  88=REFUSED  99=DON’T KNOW |  |
| P39c | ENTER TYPE OF TIME PERIOD USED IN P39b?  1=DAYS  2=WEEKS  3=MONTH(S)  4=REFUSED  5=DON’T KNOW |  |
| P40 | INTERVIEWER : WHAT IS THE RESPONDENT’S ABILITY TO COMMUNICATE (UNDERSTAND AND SPEAK) IN ENGLISH. CODE WITHOUT ASKING  1= NO DIFFICULTY  2= SOME DIFFICULTY  3= A LOT OF DIFFICULTY |  |
